# Bidirectional Crosstalk Between Cancer Stem Cells and Immune Cell Subsets

**DOI:** 10.3389/fimmu.2020.00140

**Published:** 2020-02-05

**Authors:** Luise Müller, Antje Tunger, Ioana Plesca, Rebekka Wehner, Achim Temme, Dana Westphal, Friedegund Meier, Michael Bachmann, Marc Schmitz

**Affiliations:** ^1^Faculty of Medicine Carl Gustav Carus, Institute of Immunology, TU Dresden, Dresden, Germany; ^2^National Center for Tumor Diseases, Partner Site Dresden, Dresden, Germany; ^3^German Cancer Consortium (DKTK), Partner Site Dresden, and German Cancer Research Center, Heidelberg, Germany; ^4^Department of Neurosurgery, Section Experimental Neurosurgery and Tumor Immunology, University Hospital Carl Gustav Carus, TU Dresden, Dresden, Germany; ^5^Department of Dermatology, University Hospital Carl Gustav Carus, TU Dresden, Dresden, Germany; ^6^Department of Radioimmunology, Institute of Radiopharmaceutical Cancer Research, Helmholtz Center Dresden-Rossendorf, Dresden, Germany

**Keywords:** tumor microenvironment, cancer stem cells, macrophages, T cells, myeloid-derived suppressor cells

## Abstract

Cancer stem cells (CSCs), also known as tumor-initiating cells, are characterized by an increased capacity for self-renewal, multipotency, and tumor initiation. While CSCs represent only a small proportion of the tumor mass, they significantly account for metastatic dissemination and tumor recurrence, thus making them attractive targets for therapy. Due to their ability to sustain in dormancy, chemo- and radiotherapy often fail to eliminate cancer cells with stemness properties. Recent advances in the understanding of the tumor microenvironment (TME) illustrated the importance of the immune contexture, determining the response to therapy and clinical outcome of patients. In this context, CSCs exhibit special properties to escape the recognition by innate and adaptive immunity and shape the TME into an immunosuppressive, pro-tumorigenic landscape. As CSCs sculpt the immune contexture, the phenotype and functional properties of the tumor-infiltrating immune cells in turn influence the differentiation and phenotype of tumor cells. In this review, we summarize recent studies investigating main immunomodulatory properties of CSCs and their underlying molecular mechanisms as well as the impact of immune cells on cancer cells with stemness properties. A deeper understanding of this bidirectional crosstalk shaping the immunological landscape and determining therapeutic responses will facilitate the improvement of current treatment modalities and the design of innovative strategies to precisely target CSCs.

## Introduction

In recent years, the role of the tumor microenvironment (TME) has gained an increasing amount of attention, allowing the discovery of new concepts and development of novel therapeutic approaches. Especially immune checkpoint inhibitors emerged as a promising tool to use the power of pre-existing tumor-specific T cells to target cancer cells (1, 2). Despite these advances, a significant fraction of patients fails to respond to those therapies or develops resistance and even patients with a complete response often present with recurrence and metastatic lesions later on. While there are several factors determining the response to therapy and long-term outcome, cancer stem cells (CSCs) are considered to play an important role in the resistance, metastasis, and recurrence of tumors (3). These cells, also known as tumor-initiating cells, exhibit an enhanced capacity for self-renewal, multipotency, and tumorigenicity and were first described in acute myeloid leukemia (4). Subsequently, CSCs were also identified in a multitude of solid cancers and further characterized by various cell surface markers (5). The most common markers are CD44 and CD133, with CD44 being utilized to isolate CSCs from breast, prostate, gastric, as well as head and neck squamous cell cancer (HNSCC). CD133 on the other hand is widely used to identify CSCs in glioblastoma, lung cancer, and sarcomas. Recently, more markers have been established, for example CD90 for breast cancer and glioblastoma, CD117 for breast, ovarian, lung cancer, and glioblastoma, and CD29 for breast and colon cancer (6). Importantly, due to the high plasticity of CSCs and the shared marker expression on other cells, none of these molecules is sufficient to be used as a standalone marker. So far, no consensus regarding marker combinations for the characterization and isolation of CSCs has been reached, complicating the comparison of different studies and partly explaining conflicting results. Other characteristics of CSCs that can be used for their identification are an increased activity of aldehyde dehydrogenase (ALDH) and the high expression of efflux pumps, which allows discrimination based on the exclusion of vital dyes (7, 8). The upregulation of efflux pumps is, together with a decreased sensitivity to apoptosis, an altered cell cycle, and DNA damage repair, associated with an enhanced resistance to chemotherapy (9). Considering all of these characteristics, the interaction between immune cells and CSCs is highly important, as they show distinct properties to shape the TME and represent an attractive target for therapy to improve the long-term outcome of patients.

## Impact of CSCs on the Functional Properties of Immune Cells

**Figure 1:**
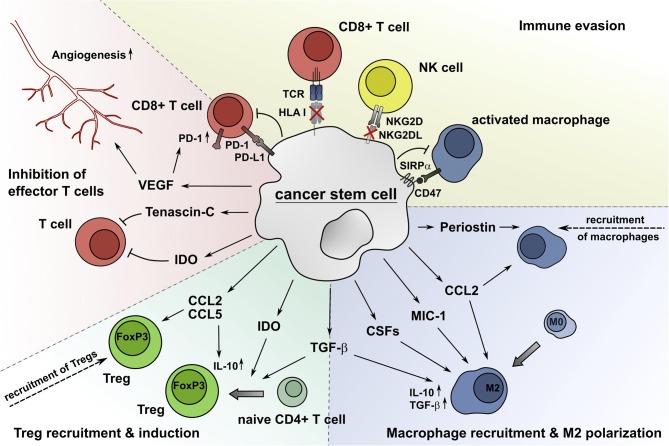
CSCs alter the composition and functional properties of immune cell subsets, which results in an impaired CSC recognition and elimination by the immune system. CSCs produce soluble molecules such as periostin, CCL2, MIC-1, CSFs, and TGF-β to attract macrophages and drive their M2 polarization. The secretion of CCL2 and CCL5 recruits Tregs into the TME, and IDO and TGF-β facilitate the generation of Tregs from naïve CD4^+^ T cells. The extracellular matrix protein tenascin-C inhibits T cell proliferation by interfering with the T cell receptor signaling pathway. Additionally, the immunomodulatory enzyme IDO impairs the expansion of effector T cells through accumulation of tryptophan catabolites, which also increases the generation of Tregs. VEGF produced by CSCs supports the induction of angiogenesis, which is critical for tumor growth, and promotes the expression of PD-1 by CD8^+^ T cells. Additionally, CSCs avoid the recognition by CD8^+^ T cells and NK cells by reducing the surface expression of HLA class I molecules and NKG2DL, respectively. The ligation of PD-L1 expressed by CSCs to PD-1 on effector T cells decreases their proliferation and IFN-γ production or leads to apoptosis. Furthermore, CSCs express the coinhibitory molecule CD47, which inhibits phagocytosis by macrophages due to repositioning of its ligand SIRPα.

Previous studies revealed the importance of the TME for both prognosis and treatment of malignant diseases and improved the understanding of the highly complex crosstalk between the TME and tumor cells (10, 11). It has been shown that tumor cells themselves can influence the immune contexture by expressing cell membrane-associated coinhibitory receptors or secreting various soluble factors to modulate certain immune subsets, shaping the TME into an immunosuppressive landscape. Due to the outstanding importance of CSCs regarding resistance, metastasis, and recurrence, their role in modulating the TME in general and major immune subsets in particular are discussed below (Figure 1).

### Macrophages

Tumor-associated macrophages (TAMs) can exhibit both pro- and antitumor properties depending on the microenvironment and their polarization. The current understanding of macrophage polarization includes the classically activated, pro-inflammatory M1 phenotype and the alternatively activated, anti-inflammatory M2 phenotype (12–14). In general, M1 macrophages exhibit anti-tumor immunity, while M2 macrophages display immunosuppressive properties and contribute to tumor progression. Therefore, a high M1/M2 ratio is associated with improved survival in multiple cancer entities (15–17). Tumor cells are known to influence the composition of intratumoral macrophages by directly recruiting M2 macrophages or driving the polarization of both tissue-resident macrophages and recruited macrophages toward a M2 phenotype (18, 19). Several studies pointed out a particular importance of CSCs in driving the recruitment of macrophages and subsequent M2 polarization. For example, Yi et al. showed that glioma-derived CSCs are associated with an increased density of intratumoral macrophages and exhibit a stronger capacity to recruit macrophages into the TME (20). Periostin, a secreted extracellular matrix protein, produced by glioma stem cells was shown to attract M2 TAMs, promoting tumor growth (21). Another study also proposed that M2 macrophages are recruited by tumor-initiating cells already at the single cell stage and that the elimination of these macrophages may abolish early tumorigenesis (22). Besides the recruitment of macrophages, CSCs are able to promote the macrophage polarization toward the M2 phenotype via secretion of various cytokines and growth factors (23). For example, the CSC-mediated M2 polarization of macrophages was shown to be driven by cyclooxygenase-2 and CCL2 in ovarian cancer (24). Similarly, glioma CSCs polarize macrophages and microglia by the production of colony-stimulating factors (CSFs), transforming growth factor β (TGF-β), and macrophage inhibitory cytokine 1 (MIC-1), and glioma CSC-conditioned medium promoted the immunosuppressive properties of macrophages (25–27). In particular, chemoresistant CSCs have a unique capacity to produce proinflammatory cytokines and soluble factors such as CCL2 and CSF-1, known to recruit and polarize macrophages (28). Altogether, this data suggests that the CSC-mediated accumulation of M2 macrophages may contribute to the generation of an immunosuppressive, pro-tumorigenic TME.

### T Cells

CSCs are also able to alter the composition and functional properties of tumor-specific effector T cells and promote the expansion of immunosuppressive, pro-tumorigenic regulatory T cells (Tregs). While a dense infiltration of CD3^+^ T cells generally correlates with a good clinical outcome in many cancers, Tregs in particular are mainly associated with a poor prognosis, with the exception of colorectal and gastric cancers (11). For example, glioblastoma CSCs were shown to produce TGF-β, promoting the induction of Tregs, and CCL2, a chemokine involved in the recruitment of Tregs (29). Furthermore, CSC-conditioned medium inhibited T cell proliferation and this effect could be reversed by blocking the signal transducer and activator of transcription 3 (STAT3) pathway. Similarly, CD44^+^ CSCs from HNSCC exhibited a significantly increased secretion of interleukin (IL)-8, granulocyte-CSF, and TGF-β compared to their CD44^−^ counterparts, suppressing the proliferation of T cells and Th1 responses while supporting the generation of Tregs (30). Another study revealed that the levels of indoleamine-2,3-dioxygenase (IDO) were particularly elevated in CSCs from breast cancer, prostate cancer, and mesothelioma cell lines, as well as primary human glioblastoma cells (31). The immunomodulatory enzyme IDO substantially suppresses T cell expansion and is involved in the generation and activation of Tregs (32). Recently, exosomes containing the extracellular matrix component tenascin-C were shown to be secreted by brain tumor CSCs, inhibiting T cell activity (33). Furthermore, You et al. proposed that ovarian CD133^+^ CSCs recruit Tregs via CCL5 and additionally enhance their immunosuppressive properties, namely the secretion of IL-10 (34). Moreover, CSCs produced elevated levels of vascular endothelial growth factor (VEGF), which is known to promote angiogenesis and increase the expression of programmed cell death 1 (PD-1) on CD8^+^ T cells (35–37). Importantly, emerging evidence suggests that the immune checkpoint molecule programmed cell death ligand 1 (PD-L1) is expressed by CSCs from glioblastoma (38), HNSCC (39), breast, and colon cancer (40, 41).

## Immune Evasion by CSCs

In addition to the modulation of the immune contexture, CSCs exhibit various properties to directly evade effector mechanisms of immune cells. The prerequisite for the elimination of tumor cells by activated CD8^+^ T cells is the presentation of peptides on the cell surface via human leukocyte antigen (HLA) class I molecules. Various molecules are involved in this process, creating a multitude of possibilities to alter the HLA class I peptide complex expression to evade the recognition by the adaptive immune system. While tumor cells in general are well-known to downregulate components of the antigen processing and presenting machinery, for example, the transporter associated with antigen processing (TAP), CSCs appear particularly specialized in this mechanism. Several groups reported a reduced expression of HLA class I or TAP molecules of CSCs in HNSCC (42), melanoma (43), glioblastoma (38), lung cancer (44), and colorectal cancer (45) in comparison to their non-stem-cell counterparts. However, Chikamatsu et al. showed a decreased expression of TAP2 in HNSCC CSCs, but were not able to find a significant difference between CD44^+^ and CD44^−^ cells with regard to other TAP molecules or the overall HLA I expression (30). Another study also failed to show a difference in the HLA class I expression of CSCs and non-CSCs in colon cancer (46). These discrepancies are likely to arise from strongly differing protocols for the isolation and culture of CSCs and also the heterogeneity of the CSC population.

In theory, missing HLA class I expression makes cancer cells more susceptible to the recognition and elimination by natural killer (NK) cells. However, conflicting results have been published, either arguing that NK cells display an enhanced recognition of CSCs, or reporting an increased evasion of NK cell-mediated killing by CSCs for example due to downregulation of activating NKG2D ligands (NKG2DL) (38, 47–50). A recent study reported that CD34^+^CD38^−^ leukemic stem cells express lower levels of NKG2DL compared to their CD34^−^ counterparts (51). Further analysis revealed that only NKG2DL^−^ AML cells exhibited chemoresistance toward cytarabine and patients with a higher proportion of NKG2DL^+^ AML cells showed a better response to chemotherapy and an improved overall survival.

Moreover, CSCs were shown to express CD47 which engages with signal regulatory protein α (SIRPα) on macrophages, inhibiting phagocytosis. Several groups reported an increased expression of CD47 on leukemic stem cells compared to non-CSCs and that the blocking of CD47 induced efficient phagocytosis (52–54). Although this mechanism seems to be predominantly found in hematologic malignancies, CD47 overexpression was also reported in lung, pancreatic, and hepatocellular carcinoma (HCC) (54–56).

## Immune Cells Drive the Formation and Maintenance of CSCs

**Figure 2:**
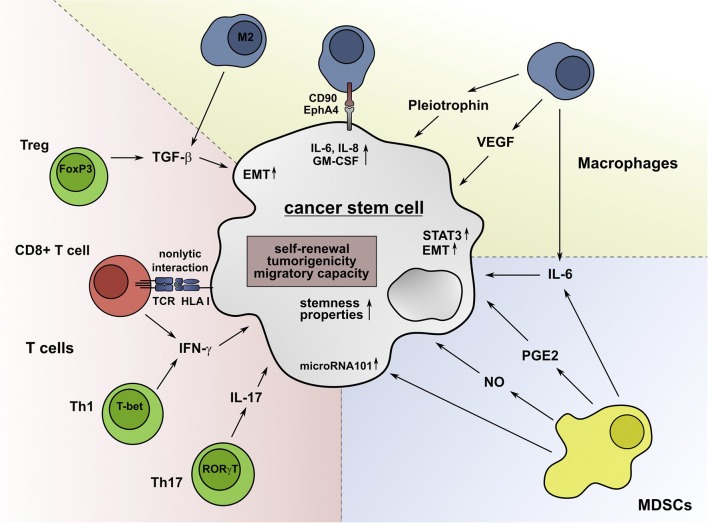
Immune cell subsets promote stemness properties in CSCs. IL-6 secreted by TAMs can convert non-stem cancer cells into CSCs and foster their drug resistance. The secretion of TGF-β by M2 macrophages can support the EMT process and the acquisition of stem-cell properties. M2 macrophages also produce VEGF, which promotes angiogenesis and the tumorigenicity of CSCs. Furthermore, the secretion of pleiotrophin by TAMs supports the CSC-driven tumor growth through activation of the Akt pathway. Besides soluble molecules, macrophages can interact with CSCs via CD90 and EphA4 in a cell-cell-contact-dependent manner, inducing the production of IL-6, IL-8, and GM-CSF. MDSCs can facilitate the expression of microRNA101 in CSCs, which increases their tumorigenicity and metastatic potential. The secretion of NO and IL-6 by MDSCs leads to a STAT3-dependent increase of the stemness properties of CSCs. Furthermore, Tregs can enhance the expression of genes, which are associated with CSCs and secrete TGF-β, which promotes the EMT and dedifferentiation of cancer cells. Moreover, Th17 cells contribute to the formation of CSCs by secretion of IL-17. Low IFN-γ levels produced by Th1 cells and CD8^+^ T cells enhance the stemness of tumor cells by activation of the Akt pathway. Non-lytic interactions of CD8^+^ T cells further promote this process by inducing the expression of genes that are associated with cancer cell dedifferentiation.

As CSCs shape the TME, infiltrating immune cells can in turn influence the characteristics of CSCs. In this chapter, the impact of major immune subsets on stemness properties, metastatic potential, and tumorigenicity of CSCs is summarized (Figure 2).

### Macrophages

While CSCs are able to recruit macrophages and promote their M2 polarization, TAMs seem to play a predominant role in the maintenance of CSCs within their niche (57). IL-6, which is mainly produced by M1 macrophages, but also by subtypes of M2 macrophages, is upregulated in breast, ovarian, prostate, pancreatic, and colorectal cancer and can confer resistance against apoptosis as well as promote proliferation, invasion, metastasis, and angiogenesis (58–64). Emerging evidence also suggests an important role for IL-6 in the induction and maintenance of CSCs. For example, IL-6 produced by TAMs supported the expansion and drug resistance of CSCs through STAT3 signaling in non-small-cell lung cancer (NSCLC) and HCC (65, 66). Moreover, multiple studies showed an IL-6-STAT3-dependent conversion of non-stem-cell breast cancer cells into CSCs, partially by promoting epithelial to mesenchymal transition (EMT) (67–69). Similarly, TGF-β secreted by M2-polarized TAMs promoted EMT and acquisition of stem-like properties in HCC (70). These findings are in line with data from Wu et al. indicating that chronic TGF-β stimulation in the course of liver cirrhosis induces expression of CSC-associated genes in liver progenitor cells and therefore promotes the development of cancer (71). Moreover, especially M2 macrophages are known to secrete VEGF, which is also produced by CSCs themselves and promotes angiogenesis, tumorigenicity, and their stem-like phenotype (72–74). Additionally, the secretion of pleiotrophin by CD163^+^ TAMs in glioma fostered CSC-mediated tumor growth and the inhibition of this pathway led to decreased tumor growth and prolonged survival in mouse xenografts (75). Besides the paracrine interaction via soluble molecules, M2 macrophages in breast cancer interact with CSCs in a cell-cell-contact dependent manner (76). The ligation of CD90 to ephrin type-A receptor 4 (EphA4) on cancer cells induced downstream signaling that resulted in the production of IL-6, IL-8, and granulocyte-macrophage colony-stimulating factor (GM-CSF), thus facilitating the maintenance of the stem-cell-like niche. In summary, these findings indicate a predominant role of macrophages in driving the induction and maintenance of CSCs.

### Myeloid-Derived Suppressor Cells

Myeloid-derived suppressor cells (MDSCs) play an important role in cancer-related immune suppression and are shown to significantly contribute to tumor progression, angiogenesis, and metastasis (77). Recently, several findings indicated that MDSCs are also involved in supporting the stem-cell features of CSCs. For example, MDSCs induce the expression of microRNA101 in ovarian cancer cells, promoting their stemness-properties and increasing the tumorigenic and metastatic potential (78). Additionally, MDSCs fostered the stemness of cervical cancer cells via the secretion of prostaglandin E2 (PGE2) (79). In breast cancer, MDSCs were shown to enhance the stem-like qualities of tumor cells by secretion of IL-6 and nitric oxide (NO) in a STAT3-dependent manner (80). Immunohistochemical analysis revealed that the presence of MDSCs positively correlated with the density of CSCs in breast cancer tissues and a high infiltration of MDSCs was associated with shorter overall survival, indicating their clinical relevance. Interestingly, STAT3 is also involved in the induction of monocytic-MDSCs in a mouse model of pancreatic cancer and monocytic-MDSCs subsequently increased the frequency of ALDH1^bright^ CSCs (81).

### T Cells

In general, tumors with a dense infiltration of CD3^+^ T cells are associated with a good clinical outcome, yet recent advances in the understanding of the immune contexture revealed various mechanisms by which infiltrating T cells can act in an immunosuppressive manner, promoting tumor progression. The most prominent mediators of immunosuppression within the T cell compartment are Tregs, exhibiting both contact-dependent and cytokine-mediated actions to inhibit effector T cells and promote tumor progression (82, 83). While CSCs are able to support Treg accumulation in the tumor, in turn, Tregs can influence the CSC-niche. For example, Treg-conditioned medium increased the side population of mouse breast cancer cell lines, supported their sphere forming capability and enhanced the expression of Sox2, Nanog, and Oct4 genes, which are associated with stemness-properties (84). Additionally, TGF-β, a hallmark cytokine of Tregs, is well-known to mediate the EMT process and therefore support the generation of CSCs (85). Besides the mechanism of EMT, a recent study described the acquisition of stem cell properties due to a TGF-β driven dedifferentiation process in colorectal cancer cells (86).

In the past years, the T helper cell subset Th17 has also been implicated in mediating not only anti-tumor activity, but also immunosuppression (87, 88). The characteristic cytokine of this subset IL-17 promoted the self-renewal of ovarian CSCs and induced stem cell features in pancreatic cancer cells (89, 90). Similarly, IL-17 was associated with an increased capacity for invasion, migration, and tumorigenicity in both *in-vitro* and *in-vivo* studies of gastric cancer cells (91). Further experiments revealed that these effects are accompanied by an increase of phosphorylated STAT3, while the results significantly decreased upon blocking the STAT3 pathway, suggesting that IL-17 acts in a STAT3-dependent manner. Importantly, these studies were conducted irrespective of the source of IL-17. Even though Th17 cells are thought to be the main producers of IL-17, some studies suggest that innate immune cells account for the majority of IL-17^+^ cells (92, 93). Additionally, hypoxia-induced expression of IL-17 by FoxP3^+^ Tregs fostered the development of CSCs in colorectal cancer, although all of these findings emerged from *in-vitro* experiments (94). Besides immunosuppressive T cell subsets and cytokines, also low doses of interferon (IFN)-γ, which is mainly produced by activated Th1 cells or CD8^+^ T cells, can increase the stemness of tumor cells in NSCLC (95). Furthermore, Stein and colleagues demonstrated that ineffective, non-lytic interactions of CD8^+^ T cells with breast cancer cells induced the expression of genes associated with stemness and dedifferentiation (96). Subsequent analysis of the generated tumors showed an increased proliferation, tumorigenicity, and capacity for metastasis.

Taken together, different T cell subsets, in addition to macrophages and MDSCs, assist CSCs to maintain their stem-cell-like state. The finding that CSCs themselves facilitate the recruitment or induction of Tregs within the tumor illustrates the strong bidirectional crosstalk between CSCs and various immune cell subsets which shapes both the TME and the CSC niche.

## Conclusion

Emerging evidence suggests that not only genetic alterations determine the development and fate of the tumor, but also the phenotype and functional properties of infiltrating immune cells. As discussed in this review, CSCs are able to shape the TME by attracting immunosuppressive cell subsets and inhibiting effector T cells. Vice versa, infiltrating immune cells interact with CSCs in various ways to promote their self-renewal, tumorigenicity, and metastasis. These findings emphasize the unique role of CSCs and the immense potential that lies in targeting them. Consequently, therapeutic strategies leading to the elimination of CSCs in addition to non-stem cancer cells may further improve the clinical outcome for tumor patients. Many of the aforementioned CSC-immune cell interactions, including the generation of M2 macrophages and MDSCs, the CSC-dependent T cell suppression, the effect of IL-6 and IL-17 on the stemness properties of CSCs, and the expression of PD-L1 are dependent on active STAT3 signaling in CSCs or immune cells. Many of these effects could be reversed by inhibition of STAT3, rendering this molecule an attractive therapeutic target to tackle both the induction of an immunosuppressive TME and the emerging consolidation of the CSC-niche (25, 29, 39, 91). For example, the STAT3 inhibitor napabucasin was shown to reduce stemness gene expression and sphere formation in different entities (97–99). Furthermore, the SIRPα ligand CD47 is overexpressed by CSCs and represents another target structure for therapy. Several studies showed an increased phagocytosis of CSCs by macrophages upon blocking of CD47 and multiple CD47 inhibitors are tested in ongoing clinical trials (53–55, 100, 101). Additionally, CSCs were shown to express increased levels of the immune checkpoint PD-L1 and PD-L1 in turn promoted the generation of CSCs, creating a rationale for combination therapies with checkpoint inhibitors (1, 102). Furthermore, TGF-β secreted by Tregs and M2 macrophages or CSCs themselves is a crucial mediator of immunosuppression that can be targeted by neutralizing antibodies or receptor kinase inhibitors (103). The inhibition of the pro-angiogenic molecule VEGF has also been proven beneficial as combinational therapy in multiple entities and could be used to disrupt both the CSC-mediated angiogenesis and the induction of stemness-properties by macrophages (104, 105). In addition to targeting the crosstalk between CSCs and the TME, CSCs can be eliminated by using specific immunotherapeutic approaches, such as drug-conjugated monoclonal antibodies, bispecific antibodies, and chimeric antigen receptor- or T cell receptor-engineered T cells, targeting antigens that are characteristically expressed by CSCs (106–109).

The described studies exploring important immunmodulatory capabilities of CSCs and the impact of various immune cell subsets on cancer cells with stemness properties led to a deeper understanding of the bidirectional crosstalk between CSCs and the immune system. However, many studies used isolated CSCs to determine their phenotype and properties, which has several limitations and can significantly influence the results (110, 111). For example, the dissociation of solid tumors usually requires enzymatic treatment, which may result in the reduction or elimination of phenotypically and/or functionally relevant CSC-associated surface molecules. The different CSC isolation procedures may also reduce the viability of the purified cells. In addition, the characteristics of CSCs in solid tumors rely on the direct interaction of CSCs with various cellular components of the TME and the extracellular matrix, which is not appropriately considered, when utilizing isolated CSCs. To circumvent these limitations, advanced technologies to explore CSCs in intact tumors, such as lineage tracing approaches, may help to gain novel insights into the phenotype and properties of CSCs and may enable the design of improved therapies to target CSCs.

## Author Contributions

LM and ATu drafted the manuscript. IP, RW, ATe, DW, FM, MB, and MS reviewed and edited the manuscript.

### Conflict of Interest

The authors declare that the research was conducted in the absence of any commercial or financial relationships that could be construed as a potential conflict of interest.
